# Genotypephenotype correlation of 17 cases of Pompe disease in Spanish patients and identification of 4 novel *GAA* variants

**DOI:** 10.1186/s13023-021-01864-8

**Published:** 2021-05-21

**Authors:** Paula Hernndez-Arvalo, Jos D. Santotoribio, Roco Delarosa-Rodrguez, Antonio Gonzlez-Meneses, Salvador Garca-Morillo, Pilar Jimnez-Arriscado, Juan M. Guerrero, Hada C. Macher

**Affiliations:** 1 Fundacin Pblica Andaluza para la Gestin de la Investigacin en Salud de Sevilla (FISEVI), Molecular Diagnosis and Rare Diseases Laboratory, Department of Clinical Biochemistry, Hospital Universitario Virgen del Roco, Seville, Spain; 2 Molecular Diagnosis and Rare Diseases Laboratory, Department of Clinical Biochemistry, Hospital Universitario Virgen del Roco, Seville, Spain; 3 Dysmorphology Unit, Department of Pediatrics, Hospital Universitario Virgen del Roco, Seville, Spain; 4 Collagenosis and Minority Diseases Unit, Experimental Cardiovascular Risk Unit, Department of Internal Medicine, Hospital Universitario Virgen del Roco, Seville, Spain; 5 Department of Clinical Biochemistry and Molecular Biology Hospital Universitario Virgen del Roco, Institute of Biomedicine of Seville (Ibis),, Seville University, Seville, Spain

**Keywords:** Pompe disease, Genotypephenotype correlation, *GAA* gene, Alpha-glucosidase enzyme

## Abstract

**Background:**

Pompe disease (PD) is an autosomal recessive metabolic disorder caused by pathogenic variants in the acid -glucosidase gene (*GAA*) that produces defects in the lysosomal acid -1,4-glucosidase. We aimed to identify genetic variations and clinical features in Spanish subjects to establish genotypephenotype correlation.

**Methods:**

A total of 2637 samples of patients who showed symptoms or susceptible signs of PD were enrolled in this observational study. Enzymatic activity was detected by fluorometric techniques and the genetic study was carried out using Next-Generation Sequencing.

**Results:**

Fourteen different variants from 17 diagnosed patients were identified, seven males and nine females with LOPD (mean age 36.07, SD 20.57, range 764) and a 2-day-old boy with IOPD, four genetic variants had not been described in the literature previously, including a homozygous variant. In all of them -glucosidase activity was decreased. Muscle weakness, respiratory distress, exercise intolerance, hypotonia, dysphagia and myalgia were commonly observed in patients.

**Conclusions:**

This study report four new genetic variants that contribute to the pathogenic variants spectrum of the *GAA* gene. We confirm that patients in Spain have a characteristic profile of a European population, with c.-32-13T>G being the most prevalent variant. Furthermore, it was confirmed that the c.236_246delCCACACAGTGC pathogenic variant in homozygosity is associated with early disease and a worse prognosis.

## Background

Pompe disease (PD) is an autosomal recessive metabolic disorder caused by pathogenic variants in the acid alpha-glucosidase gene (*GAA*) that produces biochemical defects in the lysosomal acid alpha 1,4-glucosidase. The deficient activity of the enzyme leads to lysosomal accumulation of glycogen in all tissues, especially in skeletal muscle. PD is a disorder that manifests a clinical spectrum that varies regarding the age of onset, the rate of disease progression, and the degree of organ involvement; and in general, there is an inverse correlation between the severity of the disease and the level of residual enzyme activity [1, 2]. Because of the variation in the phenotypes, PD is classified into infantile-onset Pompe disease (IOPD) and late-onset Pompe disease (LOPD). The main difference between IOPD and LOPD is cardiac findings in the first year of life. IOPD is characterized by onset during the first year of life with hypertrophic cardiomyopathy, generalized muscle weakness, hepatomegaly and respiratory dysfunction. LOPD is characterized by onset in childhood or adulthood and the presence of slow progressive muscle weakness predominantly below the waist, cardiomyopathy and respiratory distress. However, LOPD could be detected in childhood if we could use better clinical diagnostic methods such as genetic tests as newborn screening tests. The incidence of the disease varies in different ethnic groups and for different clinical forms. It was reported that the incidence of PD in Caucasian is 1:100,000 and 1:60,000 in IOPD and LOPD respectively [3]. It is not clear how many cases exist in Spanish population, the frequency of genetic variants or whether there are characteristic variants of a certain region [4].

The *GAA* gene (OMIN 606800) is situated on chromosome 17 and contains 20 exons and 19 introns extended over a distance of 20Kb. The first exon is noncoding and the beginning of the start codon is at position 33 of exon 2. To date, over 560 variants have been described in the *GAA* gene. Most of the variants described are marked as pathogenic and some of them were classified as uncertain significance. Some pathogenic variants are widely found in certain populations. For instance, the intronic variant c.-32-13T>G is the most common in Caucasian population [5] In Asian population the most common variants were c.1935C>A and c.2238G>C in Taiwanese and Chinese individuals [6], c.1316T>A and c.1857C>G in Korean individuals and c.2560C>T is the most frequent in Afro-American individuals [7]

We report here an observational study as a result of a biochemical and genetic analysis of subjects suggestive of PD. We analyzed clinical manifestations, acid -glucosidase activity and *GAA* Pompe variants of Spanish patients with Pompe disease to establish genotypephenotype correlation.

## Materials and methods

### Design of the study and patients

In this observational study, clinical and biochemical aspects and *GAA* gene sequence in a large cohort of patients from different Spanish hospitals were analyzed.

Patients included either had a family member with PD or presented more than one sign or symptom associated to PD: generalized muscle weakness, CK elevations, exercise intolerance or pain, hipotonia, hypertrophic cardiomyopathy, respiratory distress, dysphagia, dyspnea. Enzyme activity in Dried blood spots (DBS) was measured for each patient. Patients with decreased enzyme activity in DBS underwent lymphocyte determination to confirm the enzyme diagnosis. The sequencing of the *GAA* gene was performed in the patients who showed low enzyme activity in DBS and lymphocytes or who had a family member with PD.

Informed consent was signed by all patients and the study was approved by the Ethics and Research Committee of the Virgen Macarena and Virgen del Roco University Hospital (Code: 0826-N-15).

### Biochemical analysis

The determination was carried out according to the technique described by Chamoles et al. [5]. Acid -glucosidase activity was measured in DBS samples or isolated lymphocytes using 4-Methylumbelliferyl-a-D-glucopyranoside as sustrate and acarbose as inhibitor of competing enzymes at pH 4 [8, 9].

A standard curve of 4-methylumbelliferon was created to establish a relationship between the intensity of the fluorescence and the enzymatic activity as umol/L/h in DBS [cut off<0.75mol/L/h] and nmol/min/mg protein in lymphocytes (cut off<0.15nmol/min/mg protein). The cutoffs were established taking into account previous studies in our laboratory. Enzyme activity was measured in healthy and diseased subjects and statistical analysis of the data was performed. In healthy subjects, the results were normal mean 1.35mol/L/h and standard deviation 0.69 in DBS and normal mean 1.35nmol/min/mg protein and standard deviation 0.62 in lymphocytes. In subjects with Pompe disease, the results were normal mean 0.42mol/L/h and standard deviation 0.2 in DBS and normal mean 0.033nmol/min/mg protein and standard deviation 0.029 in lymphocytes.

### Molecular and bioinformatics analysis

Genomic DNA was isolated from whole blood by standard procedures using *MagNA Pure Compact Nucleic Acid Isolation Kit I. *(*Roche Diagnostics, Basle, Switzerland*). Genetic study was carried out by Next-generation sequencing (NGS). All coding regions and classical splicing sites of *GAA* gene were amplified using a custom design kit for Ion AmpliSeq in a S5 Ion Torrent Platform. The reads were aligned to Genome Reference Consortium Human Build 37 GRCh37. The limitations of the technique include non-detection in the intronic regions of the gene, nor highly repeated regions or other structural variants as inversions, translocations, large insertions or deletions.

Obtained sequences were compared with the *GAA* reference sequence NM_000152.3 to identify genetic variants. All of them had a minimum read depth of 20. Single nucleotide changes, insertions or deletions were compared to the online genome databases ClinVar and Human Gene Mutation Database (HGMD) open access and to the Erasmus MC University Medical Center Rotterdam.

After analyzing the numerous variants obtained, those with a frequency greater than 1% in the general population were discarded according to the polymorphism database [http://www.ncbi.nlm.nih.gov/projects/SNP/]. Variants that were described as benign, probably benign or polymorphisms in databases were not further researched. Novel missense variants effects were analyzed using in silico tools by Mutation Taster [http://www.mutationtaster.org] and Polyphen2 software programs [http://genetics.bwh.harvard.edu/pph2]. New variants were also analyzed using the VarSome genomic interpreter (https://varsome.com/). Novel nonsense variants that generates a premature stop codon upstream of another known disease causing nonsense variants or that affects the active protein center were evaluated as pathogenic.

## Results

**Fig. 1:**
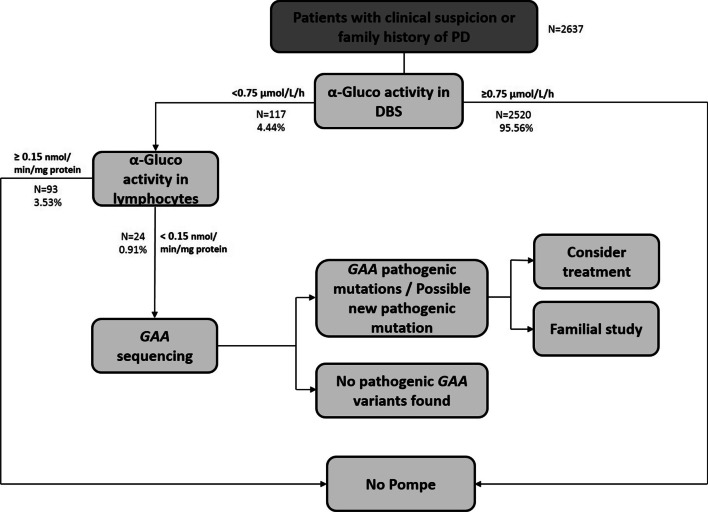
A description of PD screening protocol

From August 2016 to December 2019 were tested 2637 samples of 1343 males and 1294 females (mean age 45.16years, SD 20.77, range 098). Patients with positive screening in DBS (activity<0.75mol/L/h) are asked for a sample of lymphocytes to measure the enzyme activity and confirm the diagnosis (activity<0.15nmol/min/mg protein). The *GAA* sequence was performed in all patients whose enzyme activity measured in DBS and lymphocytes was decreased (Fig.1).

### Enzymatic acid -glucosidase activity

Of the 2637 patients studied, 2520 (95.56%) showed normal acid -glucosidase activity in DBS. The determination of the enzyme activity in lymphocytes was carried out in the 117 (4.44%) patients who showed low activity in DBS to confirm the low enzyme activity. The analysis of the enzyme activity in lymphocytes was normal in 93 (3.53% of total studied and 79.5% of 117 positives in screening) patients and a total of 24 (0.91% of total studied and 20.5 of 117 positives in screening) patients resulted with confirmed reduced activity and they were all sequenced.

### GAA genetic variants

In relation to 24 sequenced, 17 patients (#1 to #17), presented with two variants compatible with disease: seven males and nine females with LOPD (mean age 36.07, SD 20.57, range 764) and a 2-day-old boy with IOPD. Patients #18 and #19, a woman and a man respectively, showed a single variant in heterozygosity (#18 and #19) and in 5 subjects with border-line enzymatic activity no genetic justification was found in this study, their only symptom was generalized muscle weakness and they were not considered Pompe patients, it was recommended to look for other causes of myopathy. All the variants found in the molecular study were consulted in the bibliography and public databases all the variants and the unregistered ones were analyzed when possible using in silico tools.

Of all the patients who showed two variants compatible with PD, 4 subjects were homozygous and the rest had two variants in heterozygosity; it was not possible to verify whether they were in compound heterozygosity because the family study was not provided. The clinical manifestations, biochemical analysis data and genotype of the 17 patients from 16 families are summarized in Table 1. Sixteen different variants were detected and the frequency in our population is shown in Table 2. The variants found were 10 missense variants (11/36; 30.5%), one nonsense variant (1/36; 2.8%), 2 frameshifts by deletions (4/36; 11.1%), one frameshift by insertion (2/36; 5.5%) and 2 splicing variants (18/36; 50%).

**Table 1:** Clinical and biochemical analysis information of 17 patients of PD and 2 individuals with a single variant

Subject	Age	Sex	Symptoms or signs	-Glucosidase activity			
				DBS	Lymphocytes	Variant 1	Variant 2
				NR:>0.75mol/Lh	NR:>0.15nmol/m.n/mgprot		
#1	0	M	1, FM	0.5	N/A	c.236_246delCCACACAGTGC	c.236_246delCCACACAGTGC
#2	7	F	A, FM	0.12	0.01	c.-32-13T>C	c.1396_1397insG
#3	9	F	A, FM	0.15	0.07	c.-32-13T>C	c.1396_1397insG
#4	11	F	1, 2, 3	0.16	0.03	c.-32-13T>C	c.1831G>A
#5	17	F	2, 3, 4, B	0.37	0.02	c.1328A>T	c.1328A>T
#6	19	M	A, F	0.37	0.05	c.-32-13T>C	c.281_282delCT
#7	31	M	2,3	0.27	0.05	c.-32-13T>C	c.1655T>C
#8	32	F	2,3	0.3	0.09	c.-32-13T>C	c.925G>A
#9	44	F	1,8	0.34	N/A	c.-32-13T>C	c.2819C>A
#10	46	M	3, 4, 5	0.36	0.00	c.-32-13T>C	c.236_246delCCACACAGTGC
#11	51	M	2, 3, 6, 7	0.21	0.04	c.-32-13T>C	c.2104C>T
#12	52	M	3,8	0.48	0.01	c.-32-13T>C	c.1889-1G>A
#13	57	F	3, 4, 9	0.37	0.01	c.-32-13T>C	c.2237G>C
#14	58	F	3,5	0.33	0.10	c.-32-13T>C	c.-32-13T>C
#15	58	M	2,3,4	0.31	0.11	c.-32-13T>C	c.655G>A
#16	59	M	2, 3, 8	0.17	0.01	c.-32-13T>C	c.-32-13T>C
#17	64	M	2, 3, 6, 7	0.54	0.10	c.-32-13T>C	c.875A>G
#18	79	F	A, FM	0.65	0.11	c.854C>G	
#19	63	M	2, 3	0.47	0.06	c.2065G>A	

F, female; M, male; B, biopsy; FM, family member; A, asymptomatic; 1, cardiomyopathy; 2, hyperkalemia; 3, muscle weakness; 4, exercise intolerance; 5, hypotonia; 6, myalgia; 7, dysphagia; 8, respiratory distress; 9, dyspnoea

**Table 2:** Type and frequency of *GAA* variants of the patients

Type of variants	Nucleotide change	Effect on protein	Location	Frequency
Missense	c.655G>A	p.Gly219Arg	Exon 3	1/36
	c.854C>G	p.Pro285Arg	Exon 4	1/36
	c.875A>G	p.Tyr292Cys	Exon 5	1/36
	c.925G>A	p.Gly309Arg	Exon 5	1/36
	c.1328A>T	p.Asp443Val	Exon 9	2/36
	c.1655T>C	p.Leu552Pro	Exon 12	1/36
	c.1831G>A	p.Gly611Ser	Exon 13	1/36
	c.2104C>T	p.Arg702Cys	Exon 13	1/36
	c.2065G>A	p.Glu689Lys	Exon 15	1/36
	c.2237G>C	p.Trp746Ser	Exon 16	1/36
Nonsense	c.2819C>A	p.Ser940Ter	Exon 20	1/36
Deletion or insertion	c.236_246del	p.Pro79fsArgfs*13	Exon 2	3/36
	c.281_282delCT	Pro94Argfs*51	Exon 2	1/36
	c.1396_1397insG	p.Val466fs*39	Exon 9	2/36
Splicing variant	c.-32-13T>C		Intron 1	17/36
	c.1889-1G>A		Intron 13	1/36

### Clinical manifestations

Among the 17 patients with PD included in the study, one patient had IOPD phenotype and 16 had LOPD phenotype. The most frequent symptoms and sings in LOPD were muscle weakness, predominantly below the waist (62.5%), followed by high CPK serum values (37,5%) and respiratory distress (25%). Cardiomyopathy, exercise intolerance, hypotonia, dysphagia and myalgia were ascertained in 12.5% of patients. Patient #2, #3 and #6 were asymptomatic at the time of assessment. They were incorporated in the study because they had a member of the family with PD.

The only patient with IOPD, a male of two days of age (patient #1) presented at birth a hypertrophic cardiomyopathy and he died shortly after receiving the sample.

The mean of *acid -glucosidase* activity in LOPD patients was 0.30mol/L/h in DBS and 0.05nmol/min/mg protein in the isolated lymphocytes. Enzyme activity in DBS of the only infantile-onset Pompe disease patient was 0.5mol/L/h and lymphocyte measurement could not be performed due to the death of the patient.

### Genotypephenotype correlations

Two pathogenic variants were the most frequent, contributing to 58% of the total alleles. The most common variant was **c.-32-13T>G**. It was detected in 15 patients (88.2%); 2 were homozygous and 13 were heterozygous. The next most frequent pathogenic variant was **c.236_246delCCACACAGTGC** which was observed in two unrelated patients (5.8%). One patient was homozygous who presented with IOPD and one heterozygous who presented with LOPD. The variants **c.1328A>T** and **c.1396_1397insG** were identified in one homozygous and two heterozygous patients respectively. The rest of the variants (**c.281_282delCT; c.655G>A; c.875A>G; c.925G>A; c.1655T>C; c.2104C>T; c.2237G>C**) were detected once in each patient who presented with them. The total number of genetic variants detected were analyzed by bibliography and in silico predictive tools (Table 3).

**Table 3:** Analysis of variants by bibliography and in silico predictive tools

Nucleotide change	Effect on protein	ClinVar	Mutation taster	PolyPhen-2	VarSome	HGMD	Pompe database
*Described mutations*							
c.-32-13T>G		Pathogenic			Pathogenic	33	Potentially mild
c.236_246del	p.Pro79fsArgfs*13	Pathogenic	Disease causing		Pathogenic	34	Very severe
c.281_282delCT	Pro94ArgfsTer51	Likely pathogenic	Disease causing		Pathogenic		
c.655G>A	p.Gly219Arg	Pathogenic	Disease causing	Probably damaging	Pathogenic	35	Potentially less severe
c.854C>G	p.Pro285Arg	Pathogenic	Disease causing	Probably damaging	Likey pathogenic	36	Potentially mild
c.875A>G	p.Tyr292Cys	Pathogenic	Disease causing	Probably damaging	Pathogenic	37	Potentially mild
c.925G>A	p.Gly309Arg	Pathogenic	Disease causing	Probably damaging	Pathogenic	38	Potentially less severe
c.1396_1397insG	p.Val466fs*39	Not described	Disease causing		Pathogenic		
c.1655T>C	p.Leu552Pro	Pathogenic	Disease causing	Probably damaging	Pathogenic	39	Potentially less severe
c.2065G>A	p.Glu689Lys	Conflicts of interpretation	Polymorphism	Likely bening	Benign	40	
c.2104C>T	p.Arg702Cys	Pathogenic	Disease causing	Probably damaging	Pathogenic	41, 42	Potentially less severe
c.2237G>C	p.Trp746Ser	Not described	Disease causing	Probably damaging	Pathogenic	43	Potentially less severe
*Novel mutations*							
c.1328A>T	p.Asp443Val	Not described	Disease causing	Probably damaging	Likely pathogenic		
c.1831G>A	p.Gly611Ser	Not described	Disease causing	Probably damaging	Pathogenic		
c.1889-1G>A		Not described	Disease causing		Pathogenic		
c.2819C>A	p.Ser940Ter	Not described	Disease causing		Likely Pathogenic		

Four of the 14 different variants identified had not been reported previously and we considered them as likely pathogenic (**c.1328A>T, c.1831G>A, c.2819C>A, c.1889-1G>A**).

**Fig. 2:**
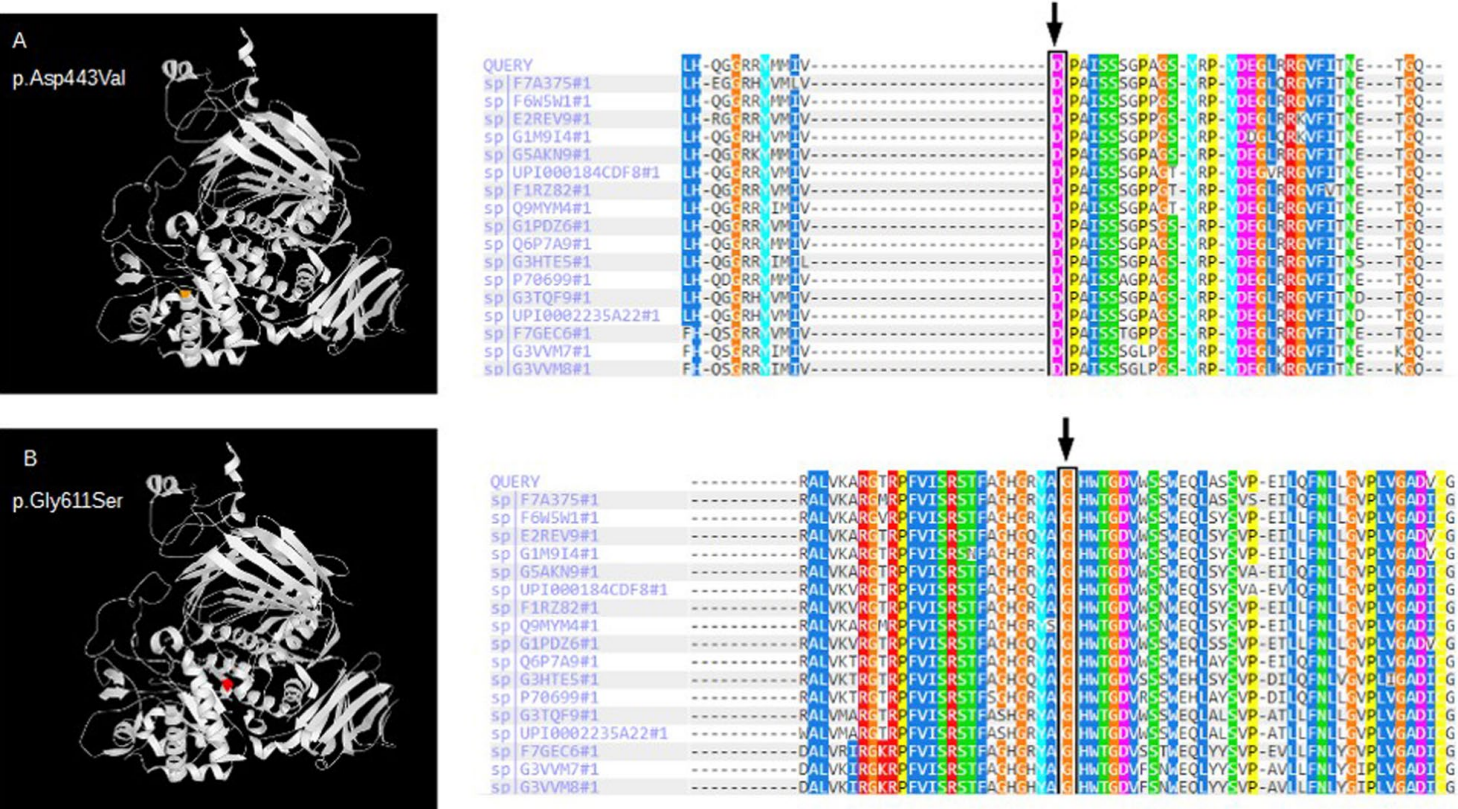
Evolutionary conservation of amino acids by missense variants across different species and position of the residues involved in the structure of human *GAA*. A: variant c.1328A>T; B: variant c.1831G>A

The missense variant **c.1328A>T** was found in homozygosity in patient #5. It results in a protein change (**p.Asp443Val)**. All of in silico tools consulted predicted a damaging effect of the variant on the protein function. Evolutionary conservation of amino acid and the position of the residue involved in the structure of the protein is showed in Fig.2. This variant was observed in homozygosity in a 17 years old woman of Pakistani origin who showed exercise intolerance, muscle weakness and high CPK in serum and also high values of glutamyl oxaloacetic transaminase and glutamyl pyruvic transaminase. She also had a muscle biopsy compatible with PD. Enzymatic activity measured in DBS was 0.37mol/L/h (V.N:>0.75mol/L/h) and 0.02nmol/min/mg protein in lymphocytes.

The missense variant **c.1831G>A** was found in heterozygosity in patient #4 (-32-13T>G+c.1831G>A). It produces a change in the protein **[p.Gly611Ser]**. The analysis with in silico predictive tools showed a probably damaging effect on the protein function. Evolutionary conservation of amino acid and the position of the residue involved in the structure of the protein is showed in Fig.2. Patient #4 is an 11 years old female who presents proximal muscle weakness, high CPK serum values and an electromyography study that showed myopathic pattern. Enzymatic activity measured in DBS was 0.16mol/L/h [V.N:>0.75mol/L/h] and 0.03nmol/min/mg protein in lymphocytes.

The novel nonsense variant **c.2819C>A** was detected in heterozygosity in patient #9 (c.-32-13T>G+c.2819C>A). It leads to a premature stop codon in protein synthesis [**p.Ser940Ter**]. Pathogenicity of the novel variant was also predicted by in silico analysis. Patient #9 is a 44years old female who presents mild left ventricular hypertrophy, muscle weakness, respiratory distress and mild hypertransaminasemia values. Enzymatic activity measured in DBS was 0.34 umol/L/h (V.N:>0.75mol/L/h).

The splicing variant **c.1889-1G>A** was observed in heterozigosity in patient #12 (-32-13T>G+c.1889-1G>A). Bioinformatics analysis were performed using Human Splicing Finder which reported that the variant disturbs the wild type acceptor site probably affecting the splicing. The other variant found was the c.-32-13T>G, widely described as a pathogenic variant. Patient #12 is a 52years old man who presents severe clinical manifestations: exercise intolerance, generalized muscle weakness and a damage to the phrenic nerve with bilateral diaphragmatic paralysis resulting in respiratory compromise. Enzymatic activity was also measured on DBS and lymphocytes (0.48mol/L/h and 0.01nmol/min/mg protein respectively).

Variants detected in patients who showed a single variant in heterozygosity were **c.854C>G** and **c.2065G>A**. Both are missense variant and were not found in other patients. Variant c.854C>G is located in exon 4 and it is detected in an asymptomatic female. Variant c.2065G>A is located in exon 15 in a male who presents muscle weakness and high level of CPK in serum. Both have been previously reported as pathogenic and benign respectively.

## Discussion

The proportion of patients with PD from the ones on suspicion of PD in other similar studies in Caucasians population was 0.29% [10] and 2.2% [11]. In our population, we tested 2637 samples with suspicion and a total of 17 new patients of PD were found (0.64%).

Except in the case of IOPD manifested from birth with hypertrophic cardiomyopathy; the median age at diagnosis in LOPD set of patients was 38years, this is in line with other authors who maintain that clinical manifestations in LOPD may present from the first decade to the seventh decade of life and the median age at diagnosis is 38years [12, 13]. In our population, three patients were asymptomatic [patients #2, #3 and #6]. All of them were included in the study because they had a family member with PD and they probably show no clinical symptoms because they are still too young. Patient #2 and patient #3 are sisters and they are 7 and 9 years old respectively and patient #6 is a 19 years old female. The delay in the manifestation of the first symptoms has delayed the diagnosis of LOPD in asymptomatic subjects as described in previous studies which show the importance of family studies for preventive follow-up [14, 15]. Currently early diagnosis is being done in with newborn screening in Taiwan, United States and Japan since a few years [16].

The clinical symptoms in our cohort were similar to the classical findings in Pompe disease studies [17, 18]. We confirm that the most common symptom in LOPD is muscle weakness, predominantly below the waist and it was present in all patients except the asymptomatic ones because they were diagnosed presymptomatically due to prior family history of LOPD. Elevated CPK levels and respiratory distress were the next most frequent symptoms. Myalgia, dysphagia or hypotonia were less frequent symptoms in our population (12.5%). Patient #1 [2days of age], showed an hypertrophic cardiomyopathy at birth, the most frequent manifestation in IOPD as reported the literature [19]. PD presents a great clinical heterogeneity, even in patients with the same genetic variant. Therefore, the type and degree of manifestations of each individual could depend on the residual enzymatic activity and its interaction with other genetic or epigenetic factors, such as the study of intronic areas or promoter methylation patterns. We suggest carry out additional studies to identify the possibility that could be concomitant factors that hinder the breakdown of glycogen, such as the possibility of being carriers of some other glycogenosis.

In accordance with others studies, our results confirm that the pathogenic variants are distributed throughout the entire gene [20,22]. As published in the bibliography, the gene has three critical regions: exon 2, which includes start codon, exon 10 and 11 where the evolutionarily conserved catalytic site domain is contained, and exon 14 which includes a highly conserved region. Two variants of our study were detected in exon 2, none in exons 10, 11 or 14. The rest of the variants are distributed by almost all exons as shown in the Table 2. Due to the NGS boom, it is expected that more variants of uncertain significance will be explained in the future.

Sequence analysis of the complete coding region of the *GAA* gene revealed 14 different variants from 17 patients including nine missense variants (26.4%), one nonsense variant (2.9%), three deletion or insertion variants (17.6%), eighteen splicing variants (52.9%).

Similar to others studies, the splice-site variant **c.-32-13T>G** was the most frequent pathogenic variant found in our cohort. As is published in the literature, the intronic variant is the most common in Caucasian populations and it is present in 4070% of the alleles in patients affected with PD [5]. In this study, it was seen in all patients except patient #1 and patient #5 (17 alleles, 50%). Patient #13 and patient #16 presented with the variant in homozygosity. This variant is located in the 3splice region and it causes aberrant splicing of the *GAA* gene. For this reason, the splicing variant c.-32-13T>G is considered pathogenic [23,25].

The next most frequent pathogenic variant present in our population was **c.236_246delCCACACAGTGC**. It was described by Palmer [26] in a patient who presented with a severe infantile-onset Pompe disease. In concordance with the previous study, we encountered the deletion in homozygosity in patient #1. Patient #1 had a sister with diagnosed PD who died at 9 months of age and for whom we do not have the results of the genetic study, the clinical information refers to parents as carriers of the disease, but the genetic study of his parents was not sent to us. It was found too in heterozygosity in patient #10, a 46years old man. The presence of the homozygous variant could be established as providing a more serious effect or being indicative of a worse prognosis.

The pathogenic variant **c.1396_1397insG** was identified in two heterozygous patients: two asymptomatic sisters (patient #2 and #3) who showed the same genotype (c.-32-13T>G+c.1396_1397insG) very young to present the PD clinical symptoms (7 and 9years old respectively). The variant is described as cause of PD creating a frame shift starting at codon Val466 and a stop codon in 39 position downstream [27]. The rest of the variants **(c.281_282delCT; c.655G>A; c.875A>G; c.925G>A; c.1655T>C; c.2104C>T; c.2237G>C)** had already been described in the literature as pathogenic were shown only once and, therefore, were less frequent in our population.

This study contributed to the identification of four new probably pathogenic variants which had not been described previously in the literature (**c.1328A>T; c.1831G>A; c.2819C>A; c.1889-1G>A**).

The substitution **c.1328A>T (p.Asp443Val)** was detected in exon 9 of patient #5. The missense variant produces a change in the protein and replaces aspartate with valine at codon 443. There are physicochemical differences between these amino acids. Acid aspartic is neutral and polar and valine is neutral and non-polar. This could modify the conformation of the protein and affect its function. Other missense variants have been reported as pathogenic in nearby codons [28, 29]. This finding suggests that this variant contributes to disease.

The missense variant **c.1831G>A** is located in exon 13 and it was shown in heterozygosity in patient #4. This substitution (**p.Gly611Ser**) replaces glycine with serine at codon 611. Glycine is non-polar and serine is polar. These physicochemical differences can alter the structure of the protein and could affect its function. On the other hand, it is the second variant discovered in this codon. The mutation c.1832G>A (p.Gly611Asp), which also change glycine for a polar amino acids, was described in previous study and was reported as pathogenic variant [30].

We detected the nonsense variant **c.2819C>A in** patient #9. This variant generates a slightly truncated protein **(p.Ser940Ter)**. It was assumed to be deleterious since the stop codons of other proteins were detected upstream of this and were known to result in a complete loss of enzyme activity. The variant c.2741delinsCAG [p.Gln944*fs30] produces a premature stop codon in aminoacid 944, was previously described by van Gelder [31]. In patients that did not present any activity of -glucosidase. This leads us to think that a previous stop codon will also generate damage to the protein.

The splicing variant c.1889-1G>A in the intron 13 was detected in patient #11. As Anna [32] published, in general, mutations in the canonical acceptor and donor sites affect strongly conserved sequences that define exonintron boundaries. Therefore, any variants in these canonical sequences might alter interaction between premRNA and proteins involved in the intron removal.

## Conclusions

In this study fourteen genetic variants in *GAA* gene were identified, as cause of Pompe disease, including four new variants. This study confirms that patients in Spain have a characteristic profile of a European population, with c.-32-13T>G being the most prevalent variant. Furthermore, it was confirmed that the c.236_246delCCACACAGTGC pathogenic variant in homozygosity is associated with early disease and a worse prognosis. We propose to extend the genetic study in the 7 individuals without genetic justification using techniques that require the study of the intronic zones of the gene or alfa-glucosidase messenger RNA.

Our findings underscore the importance of early diagnosis and propose to accurate molecular analysis to improve genetic counseling in addition to enabling a better quality of life for patients.

## Data Availability

Not applicable.
